# Identification of Novel Biomarkers for Metastatic Colorectal Cancer Using Angiogenesis-Antibody Array and Intracellular Signaling Array

**DOI:** 10.1371/journal.pone.0134948

**Published:** 2015-08-10

**Authors:** Seyung Chung, Sami Dwabe, Yayha Elshimali, Hemlata Sukhija, Clement Aroh, Jaydutt V. Vadgama

**Affiliations:** 1 Division of Cancer Research and Training, Department of Medicine, Charles R. Drew University of Medicine and Science, 1731 120^th^ street, Los Angeles, California, 90059, United States of America; 2 David Geffen UCLA School of Medicine, University of California Los Angeles, Los Angeles, California, United States of America; University of Kentucky College of Medicine, UNITED STATES

## Abstract

Colorectal cancer (CRC) is one of the three leading causes for cancer mortality. CRC kills over 600,000 people annually worldwide. The most common cause of death from CRC is the metastasis to distant organs. However, biomarkers for CRC metastasis remain ill-defined. We compared primary and metastatic CRC cell lines for their angiogenesis-protein profiles and intracellular signaling profiles to identify novel biomarkers for CRC metastasis. To this end, we used primary and metastatic CRC cell lines as a model system and normal human colon cell line as a control. The angiogenesis profiles two isogenic CRC cell lines, SW480 and SW620, and HT-29 and T84 revealed that VEGF was upregulated in both SW620 and T84 whereas coagulation factor III, IGFBP-3, DPP IV, PDGF AA/AB, endothelin I and CXCL16 were downregulated specifically in metastatic cell lines. Furthermore, we found that TIMP-1, amphiregulin, endostatin, angiogenin were upregulated in SW620 whereas downregulated in T84. Angiogenin was downregulated in T84 and GM-CSF was also downregulated in SW620. To induce CRC cell metastasis, we treated cells with pro-inflammatory cytokine IL-6. Upon IL-6 treatment, epithelial-mesenchymal transition was induced in CRC cells. When DLD-1 and HT-29 cells were treated with IL-6; Akt, STAT3, AMPKα and Bad phosphorylation levels were increased. Interestingly, SW620 showed the same signal activation pattern with IL-6 treatment of HT-29 and DLD-1. Our data suggest that Akt, STAT3, AMPKα and Bad activation can be biomarkers for metastatic colorectal cancer. IL-6 treatment specifically reduced phosphorylation levels of EGFR, HER2 receptor, Insulin R and IGF-1R in receptor tyrosine kinase array study with HT-29. Taken together, we have identified novel biomarkers for metastatic CRC through the angiogenesis-antibody array and intracellular signaling array studies. Present study suggests that those novel biomarkers can be used as CRC prognosis biomarkers, and as potential targets for the metastatic CRC therapy.

## Introduction

Colorectal cancer (CRC) is the third most common cancer in men and the second in women worldwide, accounting for approximately 608,000 deaths worldwide [1]. Despite considerable improvement in the therapeutic modalities, over 50% of CRC patients eventually developed recurrent disease and metastasis leading to death within 5 years of diagnosis [2]. Metastasis occurs in a phase of tumor progression by metastatic variant cells that possess invasive activities characterized by increased cell migration, tissue invasion, and organ colonization. To date, the mechanisms that cause CRC metastasis are not fully understood.

Cancer specific biomarkers play important roles in cancer detection, prognosis, prevention and treatment. Over the last four decades, significant efforts have been made to characterize useful biomarkers for CRC [3–5]. However, there are no valuable biomarkers for CRC metastasis. Therefore, it is essential to identify novel biomarkers that can be used to predict the metastatic potential of CRC, serve as prognostic indicators and serve as cellular targets for the targeted-therapy on CRC. In our present study, we have utilized the angiogenesis-related antibody arrays with primary and metastatic CRC cell lines to identify and characterize the novel biomarkers associated with colorectal cancer metastasis.

Angiogenesis and the development of metastases are intrinsically connected. Experimental data suggest that tumor metastases is influenced by soluble factors secreted from the original tumor [6–8]. Herein, we investigated the angiogenesis-related protein expression profiles to identify metastasis specific angiogenesis gene set. Initially, we studied two isogenic colorectal cancer cell lines SW480 and SW620. The primary cancer cell line SW480 was derived from a Dukes’ stage B colon carcinoma obtained from a 50-year old male patient and the metastatic SW620 variant was derived from the same patient’s tumor metastasized to the lymph node [9]. Since the variation from genetic background can be largely avoided for these two cell lines, they constitute a unique model for studying colorectal cancer metastasis. We further studied another pair of primary and metastatic cancer cell lines, HT-29 and T84. T84 was derived from the CRC cells that had metastasized to the lung [10].

Several pro-inflammatory cytokines released by innate and adaptive immune cells have been shown to regulate cancer cell growth and contribute to tumor promotion and metastasis. Among these, interleukin-6 (IL-6) takes a center stage in human cancer development and metastasis. An increased expression of IL-6 has been associated with an unfavorable prognosis in patients with various types of cancers including colorectal cancer [11]. Several studies have found an increased expression of IL-6 in patients with CRC, where IL-6 levels are elevated in the serum of patients and in tumor tissue itself [12–13]. IL-6 expression was associated with tumor stage, size, metastasis and survival of patients with colorectal cancer [14]. Based on these observations, we treated colorectal cancer cells with IL-6 to induce them to become more invasive and metastatic. We monitored changes in epithelial-mesenchymal transition phenotype, stem cell marker Oct-4 and EMT driver STAT3 (signal transducer and activator of transcription 3) phosphorylation in response to IL-6 treatment in a dose dependent manner. Our study provides further evidence on CRC metastasis pathophysiology, and more importantly, the discovery of novel biomarkers and potential drug targets for metastatic CRC therapy.

## Materials and Methods

### Cancer Cell Lines and Cytokine

SW480, SW620, HT-29, RKO, T84 and DLD-1 CRC cell lines were purchased from the American Type Culture Collection (ATCC; Manassas, VA, U.S.A.). Normal human colon cell line FHC (CRL-1831) was also purchased from the ATCC. They were maintained in a monolayer culture in DMEM/F12 (Dulbecco’s modified Eagle medium) with 10% fetal bovine serum, 2.5% L-Glutamine and 0.5% Penicillin/Streptomycin throughout this study. IL-6 was purchased from Sigma (catalog number: I3268, Sigma, St Louis, MO). IL-6 was added into the cell culture at 1, 5, 10 units per ml as needed. One unit is defined as the amount of IL-6 required to induce half-maximal proliferation of T-1165 cells *in vitro*.

### Angiogenesis-related Antibody Array

Human Angiogenesis Array Kit was purchased from the R&D systems (Catalog number ARY007, Minneapolis, MN, USA). 1 x 107 cells were rinsed with 1X PBS and solubilized with 1X lysis buffer provided in the kit. The angiogenesis antibody membrane was placed in the 4-well multi-dish. 2 ml of array blocking buffer was added onto each membrane. The membrane was incubated for 1 hour on a rocking platform. After the incubation, the blocking buffer was removed. The antibody membrane was then washed twice with the 1X array wash buffer and 1.5 ml of cell lysates were placed on the membrane. The membrane was incubated for overnight at 4 degrees Celsius on an orbital shaker. The membrane was then washed with 20 mL of 1X array wash buffer and continued incubation on the orbital shaker for 5 minutes at room temperature. 2 ml of streptavidin-HRP solution was added onto the membrane. It was incubated for 30 minutes at room temperature on an orbital shaker. Next, three washes were performed with 1X array wash buffer. The membrane was washed and treated with Lumi Glo and peroxide. The Bio-rad Gel Documentation System (Bio-Rad, catalog number 170–8195, Hercules, CA, USA) was used to take detailed pictures of the array using the Quantity One software using the Chemi Doc XRS function. To quantify the arrays, we used the Image J software. All the arrays were measured three times and presented with standard deviations.

### Intracellular Signaling Arrays

PathScan Intracellular Signaling Array Kit was purchased from Cell Signaling Technology (Cell Signaling Technology, Beverly, MA; Catalog #7323). For HT-29 and DLD-1, IL-6 was pre-treated and cell lysates were prepared. Whole protein lysates were prepared using lysis buffer that was provided in the kit. 100 μl of each lysates were placed onto the membrane window of the antibody array-slide. The lysate treated slide was incubated for overnight at 4°C on an orbital shaker. The slide was then washed with 100 μl 1X array wash buffer and incubated on the orbital shaker for 5 minutes at room temperature. This procedure was repeated three more times. 75 μl of 1X Detection Antibody Cocktail was added to each of the 16 wells and covered with a sealing tape provided in the kit. It was incubated for 1 hour at room temperature on an orbital shaker. Next, three times of washes were performed and the slide was incubated for 30 minutes with 75 μl 1X HRP-linked Streptavidin. The slide was washed and treated with Lumi Glo and peroxide. The Bio-rad Gel Documentation System was used to take detailed pictures of the array using the Quantity One software using the ChemiDoc XRS function.

### Activated receptor tyrosine kinase arrays

Cancer cells were washed with PBS containing 100 μmol/L Na_3_VO_4_ and solubilized in lysis buffer. 300 μg of total protein were incubated with Human Phospho-RTK Arrays (R&D Systems, Catalog number ARY001B, and Minneapolis, MN, USA). Briefly, arrays were incubated with whole-cell lysates overnight at 4°C with shaking and washed with the supplied washing buffer. Arrays were then incubated with anti—phosphotyrosine-HRP antibodies for 2 h at room temperature on a rocking platform shaker before incubation with a chemiluminescent reagent and film exposure. The arrays were scanned with the Bio-Rad Molecular Imager Gel Doc XR system.

### Western blot analyses

Monolayer cultures of respective cell lines at an 80–90% confluence were lysed using 100 μl of RIPA buffer (Thomas Scientific). Tris-glycine (Bio-Rad) gels were loaded with 50–100 μg of lysates. After running gel electrophoresis, the gel was transferred to a nitrocellulose membrane for 2 hours. The membrane was blocked for 1 hour in 5% BSA or 5% skim milk at 4°C. The membrane was then washed 3 times with 1xTTBS and incubated overnight with the primary antibody at 4°C. Primary antibodies of coagulation factor III, IGFBP-3, DPP IV, PDGF-A, endothelin I, CXCL16, TIMP-1, amphiregulin, endostatin, angiogenin and GM-CSF were purchased from Santa Cruz Biotechnology (Santa Cruz, CA). Primary antibodies for STAT3, pSTAT3, E-cadherin, Vimentin, Oct-4, VEGF and β-actin were purchased from Cell Signaling Technology (Danvers, MA). After incubation with the secondary antibodies conjugated with horseradish peroxidase (HRP), the protein bands were developed with the chemiluminescent reagents. The blot images were taken by using the Bio-rad molecular imager gel doc XR system. Western blot protein band density was measured by Image J software and presented after normalized by the house keeping protein β-actin. Normal FHC (CRL-1831) colon cell line was used as a reference for the protein expression comparison by measuring band intensity as a 1.0.

## Results

### Specific angiogenesis signature proteins are downregulated in metastatic CRC cell lines of SW620 and T84

First of all, we chose two isogenic colon cancer cell lines SW480 and SW620 and primary cell line HT29 and metastatic cell line T84 to examine the angiogenesis protein expression profiles. As a control, normal colon cell line FHC (CRL-1831) profile was analyzed in parallel. We utilized human angiogenesis array kit (R&D systems, Minneapolis, MN) and monitored the angiogenesis-related protein expression profiles. Cell lysates were prepared from the colon cancer cells and normal colon cells, respectively. Each lysate was placed onto the array membrane, incubated overnight and detected for the expression levels of 55 proteins associated with human angiogenesis. The coordinate of 55 proteins of the angiogenesis array was presented in the S1 Table. The protein expression levels were measured by the Image J software and presented as the mean pixel density normalized by the positive controls. We found six angiogenesis proteins were specifically downregulated in both metastatic SW620 and T84 cells: Coagulation factor III, IGFBP-3, DPP IV, PDGF-AA/AB, endothelin I and CXCL16 (Table 1 and S1 Fig). On the contrary, VEGF (vascular endothelial growth factor) expression was clearly upregulated in SW620 (14 folds) and T84 (36 folds), respectively. In addition, we found that TIMP-1, amphiregulin, endostatin were upregulated in SW620 while downregulated in T84. Angiogenin was downregulated in T84 and GM-CSF was downregulated in SW620. The cellular functions of these angiogenesis-related proteins were presented in table 2. Taken together, our data suggest that there is a signature angiogenesis gene set specific to the metastatic colon cancer cells.

**Table 1:** Expression levels of angiogenesis related proteins from normal colon cell, primary colorectal cancer and metastatic cancer cells. Angiogenesis arrays were performed with FHC, SW480, SW620, HT29 and T84 cell lines. Array spots were taken and analyzed by Image J software. Expression levels were presented as a mean pixel density normalized by the positive spot references.

Cell lines	Coagulation factor III	IGFBP-3	DPP IV	PDGF-AB/BB	Endothelin I	CXCL16
FHC	2.53 ± 0.24	0.71 ± 0.08	1.35 ± 0.042	9.70 ± 1.26	0.50 ± 0.026	1.26 ± 0.63
SW480	58.36 ± 1.37	81.36 ± 4.38	7.97 ± 0.88	135.45 ± 2.45	33.92 ± 0.75	72.59 ± 1.74
SW620	28.70 ± 5.06	3.97 ± 0.05	3.76 ± 0.35	1.25 ± 0.072	9.48 ± 0.27	0.01 ± 0.020
HT29	48.25 ± 2.09	21.29 ± 2.0	93.56 ± 1.40	17.73 ± 2.03	26.2 ± 0.31	6.93 ± 1.54
T84	20.18 ± 4.02	1.18 ± 0.87	14.05 ± 0.38	0.26 ± 0.05	2.05 ± 1.11	3.79 ± 1.06
	VEGF	TIMP-1	amphiregulin	Endostatin	angiogenin	GM-CSF
FHC	0.02 ± 0.031	0.55 ± 0.049	1.36 ± 0.30	1.28 ± 0.09	5.13 ± 0.49	1.33 ± 0.53
SW480	2.23 ± 0.75	96.44 ± 2.73	0.82 ± 0.46	28.27 ± 1.3	2.44 ± 0.48	70.53 ± 4.88
SW620	28.28 ± 0.27	86.95 ± 1.38	15.27 ± 1.23	18.75 ± 2.13	0.19 ± 0.27	1.72 ± 0.33
HT29	0.35 ± 0.31	106.86 ± 3.21	103.9 ± 1.94	22.54 ± 1.11	68.24 ± 1.31	0.32 ± 0.012
T84	11.41 ± 1.11	39.10 ± 1.41	23.85 ± 1.79	3.22 ± 1.02	27.30 ± 0.20	0.25 ± 0.021

**Table 2:** The cellular functions of angiogenesis-related proteins specifically downregulated in the metastatic colorectal cancer cells.

Proteins	Functions	References
Coagulation factor III	A cell surface glycoprotein. This factor enables cells to initiate the blood coagulation cascades, and it functions as the high-affinity receptor for the coagulation factor VII.	[15]
CXCL16	Trans-membranous CXCL16 chemokine reduces proliferation while soluble CXCL16 chemokine enhances proliferation and migration.	[16]
GM-CSF (Granulocyte-macrophage colony-stimulating factor)	A protein secreted by macrophages, T cells, mast cells, NK cells, endothelial cells and fibroblasts. It is a cytokine that functions as a white blood cell growth factor. It stimulates stem cells to produce granulocytes (neutrophils, eosinophils, and basophils) and monocytes.	[17]
Endothelin 1	One of three isoforms of human endothelin (ET-1). This peptide is a potent vasoconstrictor and is produced by vascular endothelial cells.	[18]
Endostatin	A naturally-occurring, 20-kDa C-terminal fragment derived from type XVIII collagen. It is reported to serve as an anti-angiogenic agent.	[19]
IGFBP-3 (Insulin-like growth factor binding protein 3)	A protein with an IGFBP domain and a thyroglobulin type-I domain. The protein forms a ternary complex with insulin-like growth factor acid-labile subunit and either insulin-like growth factor I or II.	[20]
PDGF (Platelet-derived growth factor)	A potent mitogen for cells of mesenchymal origin, including smooth muscle cells and glial cells. PDGFs are expressed in colon cancer cells and their receptors (PDGF-Rs) are overexpressed by various stromal cell populations.	[21]
DPP IV (Dipeptidyl peptidase-4)	An antigenic enzyme expressed on the surface of most cell types and is associated with immune regulation, signal transduction and apoptosis.	[22]
TIMP-1 (TIMP metallopeptidase inhibitor 1)	A tissue inhibitor of metalloproteinases, is a glycoprotein that is expressed in many different tumors.	[23]
Angiogenin	A potent stimulator of new blood vessels through the process of angiogenesis.	[24]
Amphiregulin	An autocrine growth factor as well as a mitogen for astrocytes, Schwann cells and fibroblasts.	[25]

### Common angiogenesis gene signature exists in the metastatic CRC cell lines SW620 and T84

To identify the common angiogenesis genes specific to metastatic CRC, we have aligned antibody arrays from primary SW480 and metastatic SW620 and T84 together. We reasoned if there is a universal metastasis signature in angiogenesis, it should show the same expression pattern even from the different metastatic CRC cell lines. Isogenic primary SW480 was selected as a reference cell line. SW480 and two metastatic cell lines SW620 and T84 array comparison revealed the angiogenesis protein expression differences. We found CXCL16, GM-CSF, endostatin, endothelin-1, IGFBP-3 and PDGF/AB, BB expression levels were clearly reduced in metastatic SW620 and T84 cells.

### Western blot analyses confirmed the angiogenesis array data

**Fig 1:**
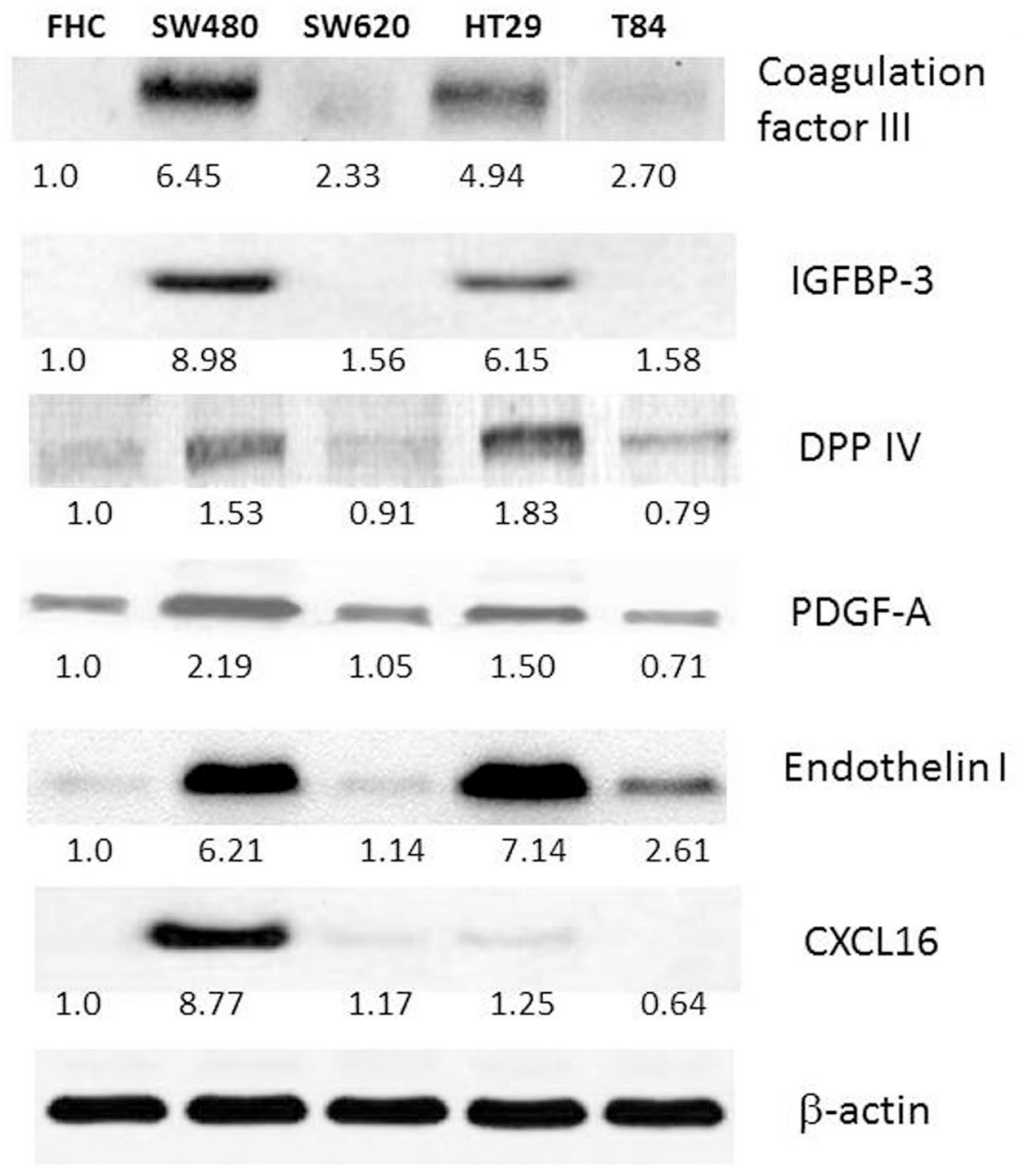
Western blot analyses of normal colon cell line FHC, primary cell line SW480 and HT-29 and metastatic SW620 and T84 for angiogenesis protein expression profiles. Western analyses were performed with the normal cell line and primary and metastatic colorectal cancer cell lines. Angiogenesis proteins identified from the array study, coagulation factor III, IGFBP-3, DPP IV, PDGF-A, endothelin I and CXCL16 were run on the gel and analyzed. Protein bands were measured for intensity and quantified using normal colon cell FHC as a reference.

**Fig 2:**
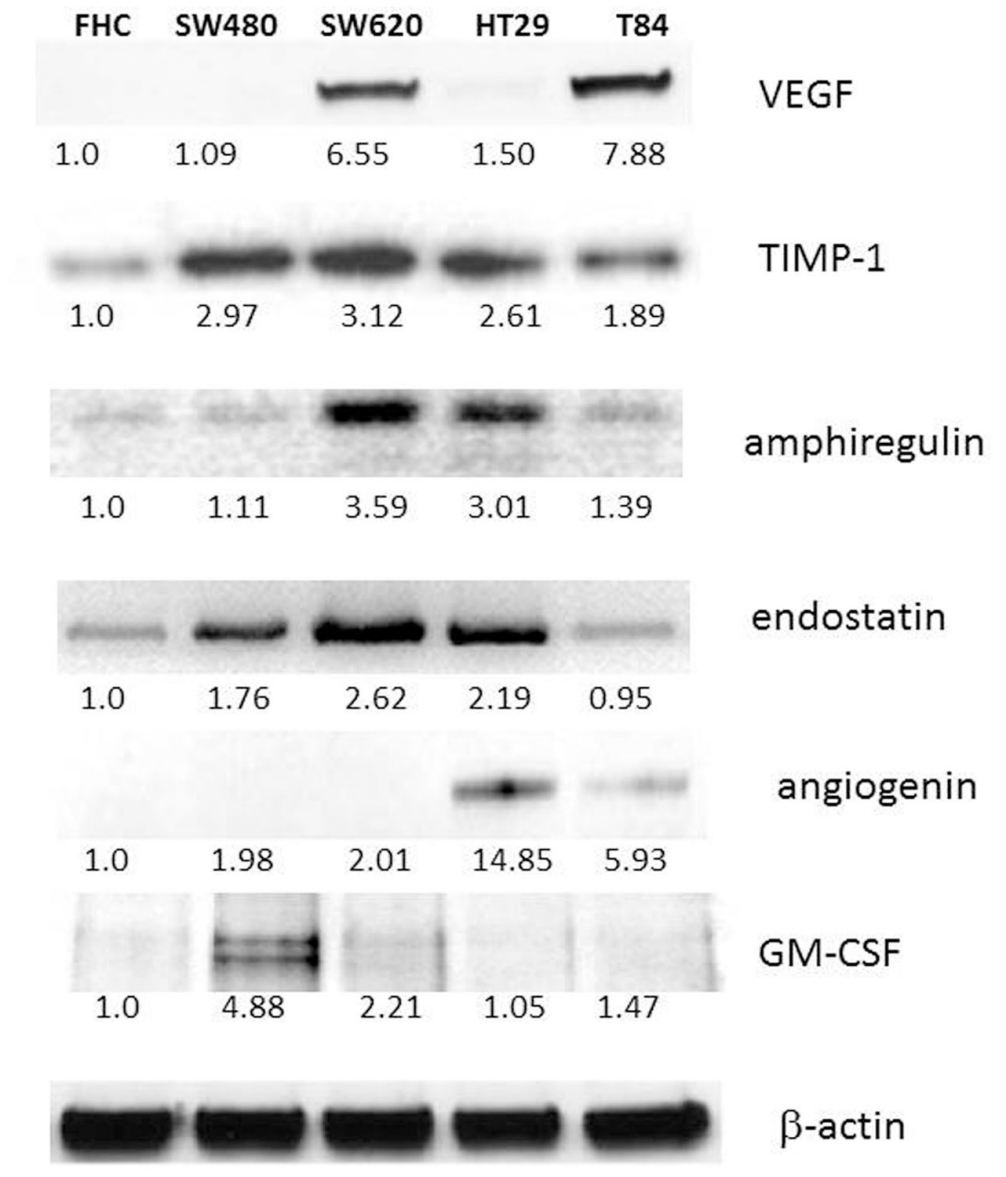
Western analyses of normal colon cell FHC, primary and metastatic colon cancer cell lines of SW480, SW620, HT-29 and T84. Immunoblotting was performed for the VEGF specifically upregulated in metastatic colon cancer and TIMP-1, amphiregulin, endostatin, angiogenin and GM-CSF. Protein expression levels were measured and normalized by the normal colon cell FHC expression.

To verify the angiogenesis array data, we performed western blots of the identified the proteins and measured the expression levels in normal and colorectal cancer cells. In agreement with the array data, coagulation factor III, IGFBP-3, DPP IV, PDGF-A, endothelin I and CXCL16 were specifically downregulated in both SW620 and T84 (Fig 1). Normal colon cell line FHC was used as a reference cell and measured as a 1.0 fold. We found all six proteins were upregulated in primary cell lines SW480 and HT29 compared to FHC, then downregulated in metastatic cell lines of SW620 and T84. Another set of angiogenesis proteins, TIMP-1, amphiregulin, endostatin were upregulated in SW620 whereas downregulated in T84 (Fig 2). This might reflect that the proteins function in organ specificity as SW620 metastasize to lymph node while T84 metastasize to lung. Angiogenin was upregulated in HT29 and downregulated in T84. Finally, GM-CSF was upregulated in SW480 whereas downregulated in SW620. Our results indicate that these angiogenesis signature genes represent the CRC metastasis and can be used as novel prognosis factor as well as cellular targets for CRC therapy.

### Cytokine IL-6 treatment induced epithelial-mesenchymal transition and Oct-4 expression from human primary CRC cell lines

Since we determined angiogenesis signature for established metastatic CRC cells, we next wished to find the cellular responsive changes to IL-6 treatment. IL-6 is regarded as an important tumor promoting factor in various types of cancers including lymphoma, melanoma as well as breast, ovarian, pancreatic, prostate and colorectal cancers. More importantly, recent studies reported that human tumors became more invasive and metastatic to both autocrine and paracrine IL-6 signaling [26]. We tested our hypothesis that IL-6 treatment may induce the primary CRC cell lines more invasive and aggressive through the epithelial-mesenchymal transition (EMT) process. Primary CRC cell lines HT-29 and DLD-1 were treated with IL-6 in a gradient of 1~ 10 units/ml for 72 hours. Cell lysates were prepared and applied to western blot analyses to monitor the EMT biomarker expression levels. We also monitored STAT3 activation simultaneously since STAT3 is known to be phosphorylated by JAK (Janus Kinase) which is activated by IL-6 triggered signal transduction [27]. Lastly, we have examined the expression of embryonic stem cell marker Oct-4. Oct-4 is one the most critical transcription factors for tumorigenesis. Oct-4 expression is also associated with cancer stem cell traits as well as tumor invasiveness.

**Fig 3:**
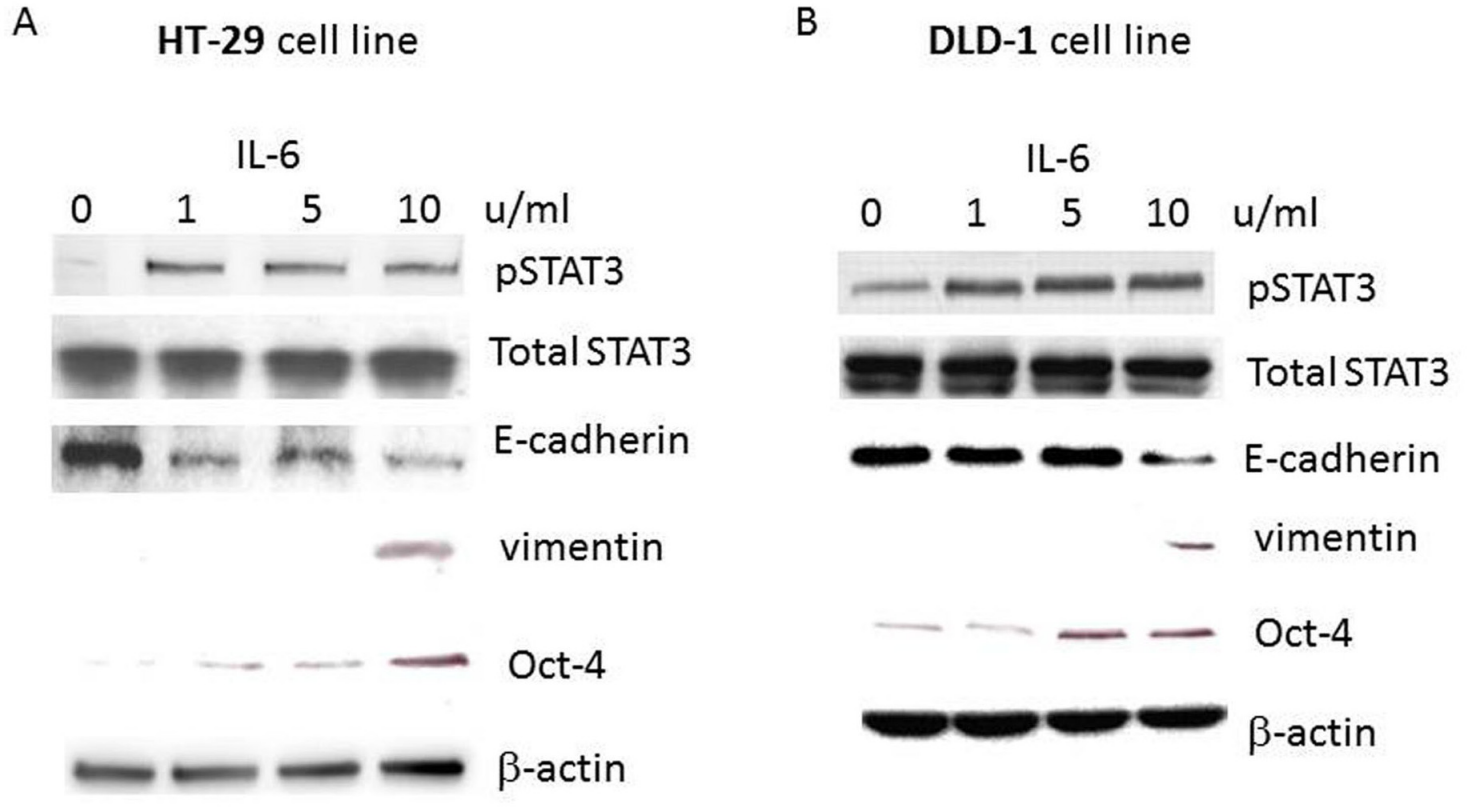
Western blot analyses of primary CRC cell lines treated with IL-6. To investigate EMT and STAT3 activation of human CRC cells, cytokine IL-6 was treated on a gradient level of 0, 1, 5 and 10 u/ml. A: HT-29 was treated with a gradient of IL-6, then STAT3 phosphorylation and EMT phenotype was observed. Another important stem cell marker Oct-4 expression was also monitored. B: DLD-1 cells were treated with IL-6 and subjected to the western analyses.

STAT3 was phosphorylated upon IL-6 treatment in a dose-dependent manner in both HT-29 and DLD-1 cell lines (Fig 3). pSTAT3 expression was enhanced from IL-6 treatment of 1 unit/ml condition whereas total STAT3 expression levels remained the same. This is consistent with the IL-6 triggered JAK mediated STAT3 phosphorylation pathway. For EMT phenotype, standard EMT biomarker E-cadherin expression was reduced while mesenchymal marker vimentin expression was elevated upon IL-6 treatment (Fig 3A and 3B). Finally, Oct-4 expression was induced in HT-29 and DLD-1 cells with the IL-6 (5u/ml) and IL-6 (10 u/ml) treatments, respectively. These results confirm that IL-6 elicits EMT process in human CRC cells. Moreover, concurrent expression of Oct-4 and pSTAT3 with IL-6 treatment data suggest the IL6-JAK-STAT3-Oct-4 signaling axis exist in CRC cells and contributes to tumor metastasis and invasiveness. In agreement with our study, Chang and associates have recently shown the increased levels of IL-6 at the leading edge of invasive human breast tumors [28]. They found the increased levels of IL-6 at the tumor leading edge and positively correlated with advanced stage, suggesting a mechanistic link between tumor cell production of IL-6 and invasion.

### Metastatic CRC cell line SW620 and IL-6 treatment of HT-29 and DLD-1 showed the same intracellular signaling activation pattern

**Fig 4:**
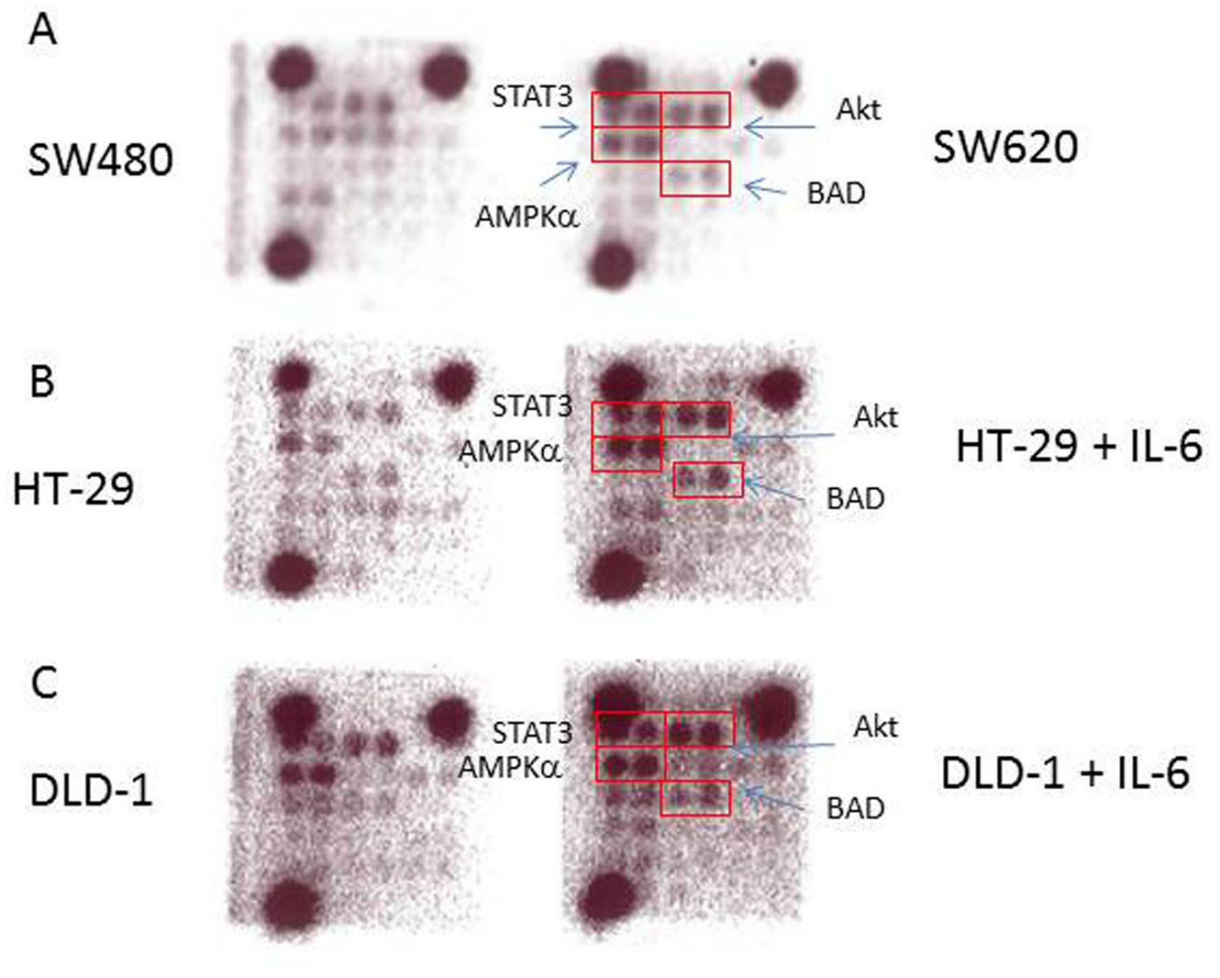
Intracellular signaling arrays of human CRC cell lines. To monitor the intracellular signaling, important signaling component activation was observed using antibody arrays. A: Two isogenic CRC cell lines, SW480 and SW620, were analyzed for the signaling activation. Phosphorylation levels of STAT3, Akt, AMPKα and BAD proteins were elevated in SW620 compared to SW480 cells. B: HT-29 was treated with IL-6 and subjected to intracellular signaling array study. Interestingly, the same protein set of STAT3, Akt, AMPKα and BAD phosphorylation levels were all increased upon IL-6 treatment. C: DLD-1 cells were treated with IL-6 and observed for the signaling component activation. DLD-1 also showed the STAT3, Akt, AMPKα and BAD phosphorylation enhancement upon IL-6 stimulation.

Since IL-6 treatment induced EMT phenotype, STAT3 activation and Oct-4 activation, we next wished to determine which intracellular signaling pathways are activated upon IL-6 treatment. To this end, we utilized PathScan Intracellular Signaling Array kit (Cell Signaling Technology, catalog number 7323) and performed the array analysis as described in Methods. The coordinates of the intracellular signaling array was also presented (S2 Table). First of all, we investigated the two isogenic SW480 and SW620 cell lines and compared the signaling activation. Cell lysates were prepared and incubated onto the intracellular signaling array membrane overnight. The following day, antibody membrane was incubated with 1X HRP-linked Streptavidin and visualized with Lumi Glo and peroxide. Images were taken using Bio-rad gel documentation system. As shown in Fig 4, phosphorylation levels of STAT3, Akt, AMPKα and BAD were elevated in SW620 compared to primary SW480 (Fig 4A). The same proteins were phosphorylated at very modest levels in SW480. Our results suggest that STAT3, Akt, AMPKα and BAD signaling pathways were enhanced in metastatic CRC cell lines.

We next wished to know if the same signaling components were activated upon IL-6 treatment on the primary CRC cell lines. Thus, we treated HT-29 and DLD-1 with IL-6 at the 10 u/ml dosage and examined the intracellular signaling pathways using the same antibody arrays. Interestingly, IL-6 treated HT-29 and DLD-1 also showed STAT3, Akt, AMPKα and BAD signaling activation on the antibody arrays (Fig 4B and 4C). Our data suggest that there exists common signature signaling activation in human CRC cells, and the signature can be used as a prognosis biomarker or targets for the CRC therapy in a clinical setting.

### Receptor Tyrosine Kinase Array revealed phosphorylation levels of EGFR, HER2, Insulin R and IGF1R were decreased upon IL-6 treatment of HT-29

**Fig 5:**
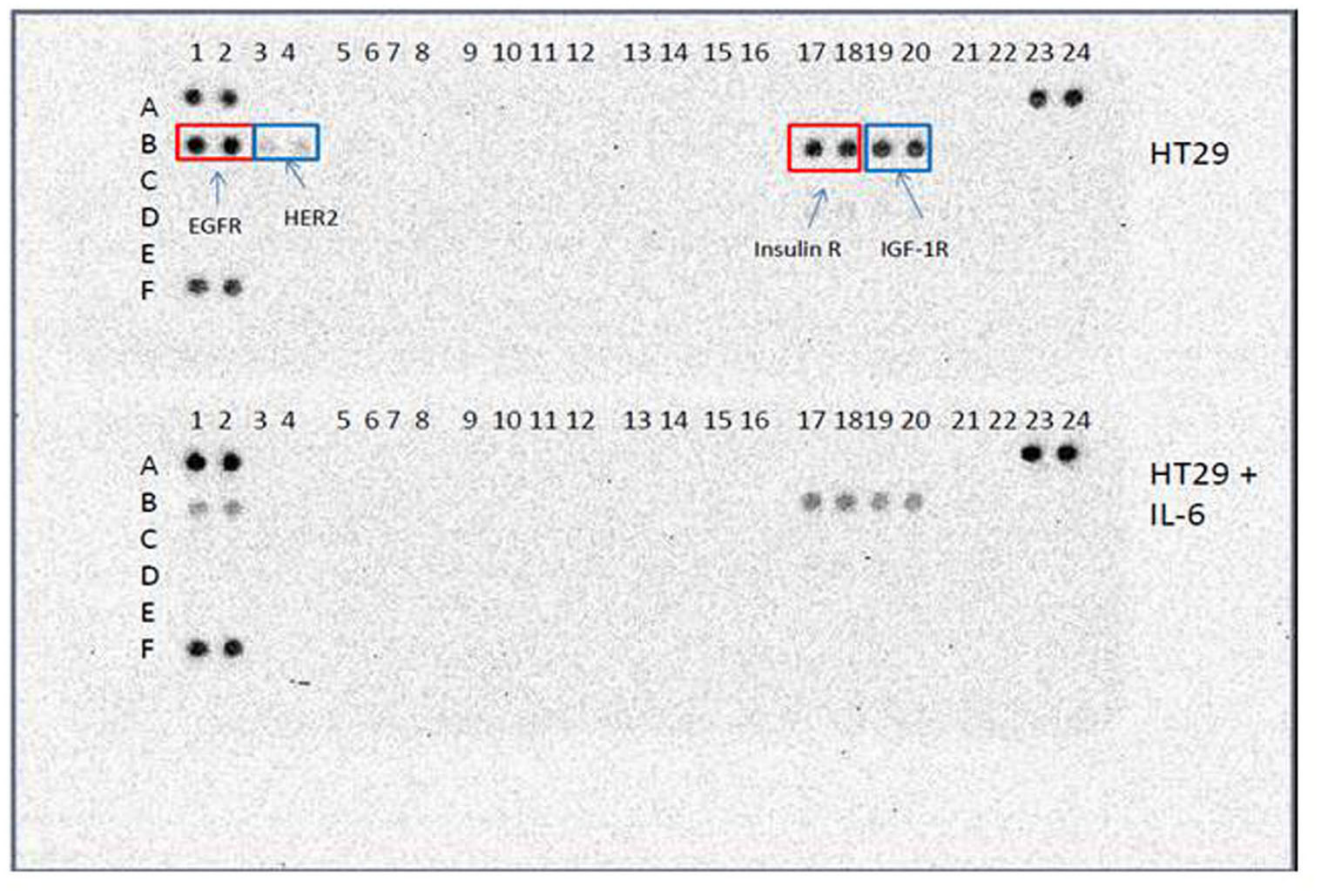
Human phosphor-receptor tyrosine kinase array of HT-29. We investigated the receptor tyrosine kinase phosphorylation with IL-6 treatment in HT-29. A: Untreated control HT-29 array showed the phosphorylated EGFR, HER2, Insulin R and IGF-1R receptors. B: When we treated HT-29 with IL-6, the phosphorylation of EGFR, HER2, Insulin R and IGF-1R was abolished.

We further investigated cancer cellular response to IL-6 treatment. Since we examined the intracellular signaling and found the signature activation, we extended our study to the receptor tyrosine kinase activation. To this end, we have used the human phosphor-tyrosine kinase array (R&D Systems, Catalog number ARY001B) kit and analyzed IL-6 effects on the human CRC cells. HT-29 cells were treated with IL-6 (10 u/ml) and lysed with lysis buffer provided. ~ 300 μg of total proteins were placed on the human phosphor-RTK array membrane and incubated overnight. The antibody membrane was incubated with secondary antibody and the positive protein spots were detected by chemiluminescence. The coordinates of this human phosphor-RTK array was also presented (S3 Table). We found that EGFR and HER2 receptors were concurrently phosphorylated in HT-29 (Fig 5A). Insulin receptor and IGF 1R receptor were also phosphorylated in HT-29. However, upon IL-6 treatment, the phosphorylation levels of EGFR, HER2 receptor, insulin R and IGF 1R receptors were clearly decreased (Fig 5B). Our data suggest that IL-6 induced EMT process is associated with downregulation of several phosphorylated tyrosine kinase receptors.

**Fig 6:**
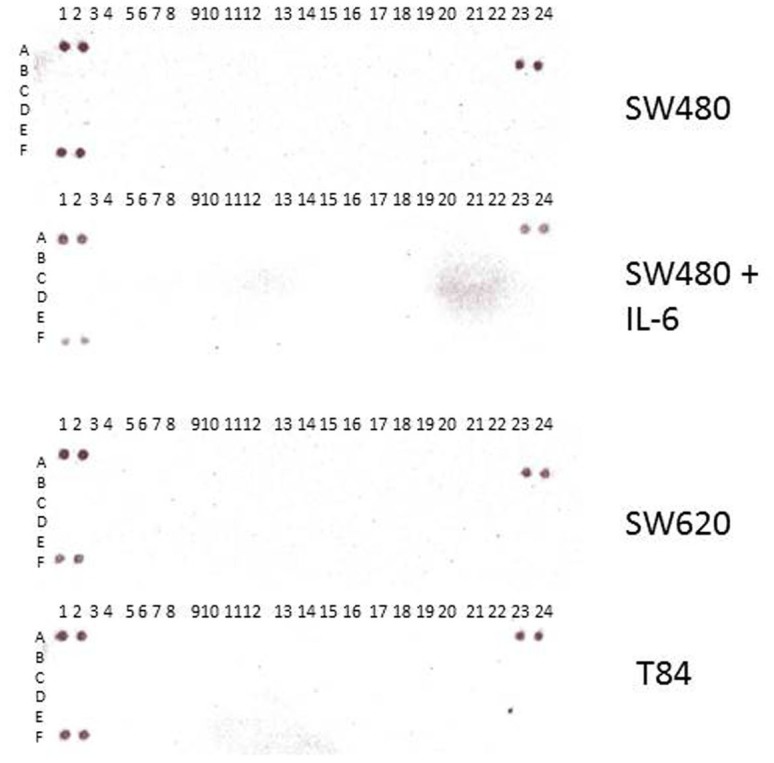
Human phosphor-receptor tyrosine kinase array of SW480, SW620 and T84. We investigated the receptor tyrosine kinase phosphorylation with IL-6 treatment in SW480. Untreated control SW480 and IL-6 treated SW480 arrays showed no RTK activation. Moreover, metastatic cancer cell line SW620 and T84 did not show the RTK activation, either.

We also investigated SW480 cells untreated and treated with IL-6, SW620 and T84 cells for the RTK profiles (Fig 6). However in those cell lines, receptor tyrosine kinases were not activated. This is likely due to the colorectal cancer cell line specificity. Analyses of more cancer cell lines remain to be seen.

## Discussion

The major cause of death from colorectal cancer (CRC) is from tumor metastasis. Almost 50% of CRC patients ultimately developed recurrent disease and metastasis leading to death within 5 years of diagnosis. Metastasis occurs in a phase of tumor progression by metastatic variant cells that possess invasive activities characterized by increased cell migration, tissue invasion, and organ colonization.

In our present study, we hypothesized that there might be a signature gene set which is selectively activated (or inactivated) in metastatic colorectal cancer. We tested this hypothesis by investigating metastatic and primary CRC cell lines for their specific angiogenesis-protein expression patterns. Our study has identified a novel gene subset specific for metastatic CRC cells. Initially we analyzed the established CRC cell lines, followed by treatment with the IL-6 cytokine to induce metastatic phenotype in human CRC cells. IL-6 elicited concurrent STAT3 activation and EMT biomarker expressions. Investigation of the intracellular signaling and receptor tyrosine kinase arrays revealed more novel gene signature activation in these CRC cells. Overall, our study identified novel biomarkers for metastatic CRC which can be used not only as a prognosis factor(s) but also as novel targets for the metastatic CRCs.

Lymph metastasis is one major concern in CRC. We chose two isogenic CRC cell lines SW480 and SW620, and uncovered angiogenesis-specific proteins that are upregulated and downregulated in lymph metastatic SW620 cells. Interestingly, when we examined lung-metastatic CRC cell line T84, we found certain angiogenesis-proteins were downregulated in both SW620 and T84 whereas other proteins were downregulated specifically to either SW620 or T84. Commonly downregulated proteins; CXCL16, GM-CSF, endothelin-1, endostatin, IGFBP-3 and PDGF-AB/BB may constitute a universal metastasis signature in CRC. Meanwhile, SW620 specific protein, for example coagulation factor III, may be a lymph node directed angiogenesis factor. Similarly, T84 specific proteins, e.g. angiogenin and amphiregulin, are likely the lung-specific metastatic angiogenesis proteins. Our results indicate that in primary colon cancer cells, PDGFs are expressed whereas in metastatic colon cancer cells, PDGF expression may be downregulated. However, the stromal PDGF signaling for metastatic cancer cells may be overexpressed in colon cancer patients. It remains to be seen if this is the case in clinical specimens of colorectal tumors.

Epithelial-mesenchymal transition (EMT), a transdifferentiation process characterized by decreased epithelial markers such as E-cadherin and increased mesenchymal markers such as vimentin have been implicated to play a role in which transformed epithelial cells acquire the ability to invade, resist apoptosis and metastasize [29]. Notably, the EMT process often results in the expression of cancer stem cell traits [30]. We have treated CRC cells with cytokine IL-6 and induced EMT phenotype concurrently with pSTAT3 and Oct-4 expression. Both autocrine and paracrine IL-6 signaling induced tumor aggressiveness. Our data are consistent with the clinical data in which IL-6 plays a role in tumorigenesis and metastasis (28). Furthermore, we have shown the specific intracellular signaling components that are activated by IL-6, STAT3, Akt, AMPKα and BAD. Finally, we discovered that specific receptor tyrosine kinase (EGFR, HER2) activities are downregulated by IL-6 in human CRC HT-29 cells. Further studies are geared toward development of inhibitor cocktails for these novel CRC biomarkers in a clinical application. In summary, our study has identified a potential pathway for targeted-therapy for CRC using the novel biomarkers for metastatic CRC.

## Supporting Information

S1 Fig Angiogenesis-related protein expression profiles of FHC, SW480, SW620, HT-29 and T84. Primary and metastatic colorectal cancer cell lines SW480, SW620, HT29 and T84 were analyzed for angiogenesis protein expression profiles. SW480 expressed coagulation factor III, CXCL16, GM-CSF, endothelin 1, endostatin, IGFBP-3, PDGF-AB/BB clearly whereas did not express VEGF. SW620 expressed VEGF whereas downregulated coagulation factor III, CXCL16, GM-CSF, endothelin 1, endostatin, IGFBP-3, PDGF-AB/BB expressions. In parallel, colorectal cancer cell lines HT-29 and T84 were analyzed for angiogenesis protein expression profiles. In HT-29. DPP IV, TIMP-1, angiogenin, endothelin, amphiregulin and PDGF/AB, BB proteins were expressed whereas VEGF was not expressed in HT-29. In T84, VEGF was expressed while DPP IV, TIMP-1, angiogenin, endothelin, amphiregulin and PDGF/AB, BB expression levels were decreased.
(JPG)

S2 Fig Human angiogenesis array alignment for metastasis signature. To identify common proteins that were upregulated or downregulated selectively in metastatic CRC cell lines, two metastatic CRC cell lines, SW620 and T84, their angiogenesis arrays were aligned with primary cell line SW480. Primary CRC cell line SW480 was used as a reference cell line, lymph-metastatic SW620 cell array was presented. In parallel, lung-metastatic T84 array was aligned. VEGF was upregulated in both SW620 and T84 cells. On the contrary, CXCL16, GM-CSF, endostatin, endothelin-1, IGFBP-3 and PDGF/AB, BB protein expression levels were decreased in both SW620 and T84 cells.
(JPG)

S1 Table Coordinates of human angiogenesis array. 55 angiogenesis related proteins were presented in the S1 Table. The coordinates and target proteins were indicated together.
(DOCX)

S2 Table Coordinates of human intracellular signaling array. 18 Intracellular signaling array proteins were presented. The coordinates and the target proteins were matched and presented.
(DOCX)

S3 Table Coordinates of human phosphor-receptor tyrosine kinase array. The human receptor tyrosine kinase proteins were presented. The coordinates and target proteins were indicated in the table.
(DOCX)
