# Supplementary figures and images for: Identification of Novel Biomarkers for Metastatic Colorectal Cancer Using Angiogenesis-Antibody Array and Intracellular Signaling Array
Source: PLoS One. 2015 Aug 10;10(8):e0134948. doi: 10.1371/journal.pone.0134948 (PMC4530953; doi:10.1371/journal.pone.0134948)

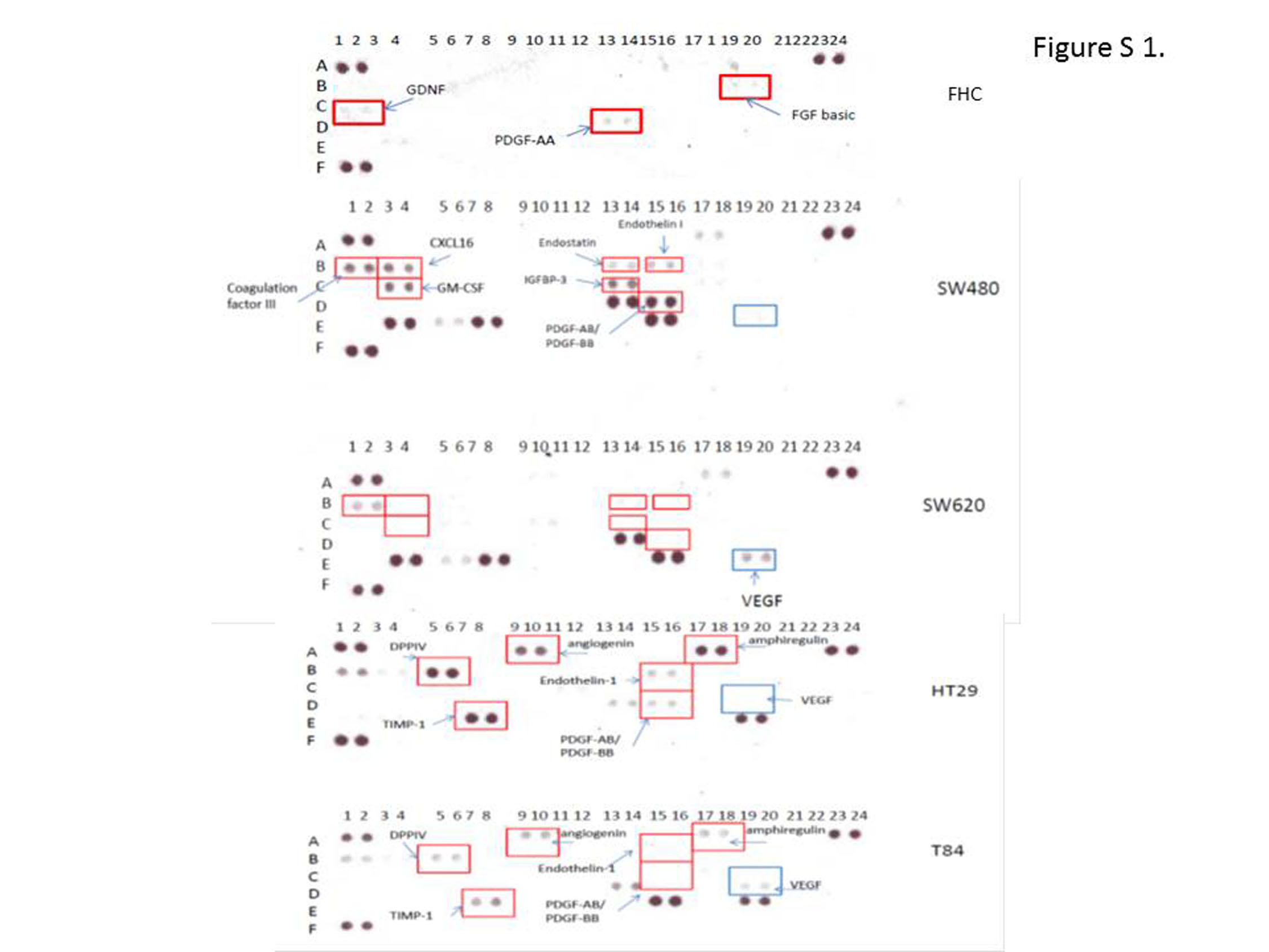

Supplement: S1 Fig — Primary and metastatic colorectal cancer cell lines SW480, SW620, HT29 and T84 were analyzed for angiogenesis protein expression profiles. SW480 expressed coagulation factor III, CXCL16, GM-CSF, endothelin 1, endostatin, IGFBP-3, PDGF-AB/BB clearly whereas did not express VEGF. SW620 expressed VEGF whereas downregulated coagulation factor III, CXCL16, GM-CSF, endothelin 1, endostatin, IGFBP-3, PDGF-AB/BB expressions. In parallel, colorectal cancer cell lines HT-29 and T84 were analyzed for angiogenesis protein expression profiles. In HT-29. DPP IV, TIMP-1, angiogenin, endothelin, amphiregulin and PDGF/AB, BB proteins were expressed whereas VEGF was not expressed in HT-29. In T84, VEGF was expressed while DPP IV, TIMP-1, angiogenin, endothelin, amphiregulin and PDGF/AB, BB expression levels were decreased. (JPG) [file pone.0134948.s001.jpg]

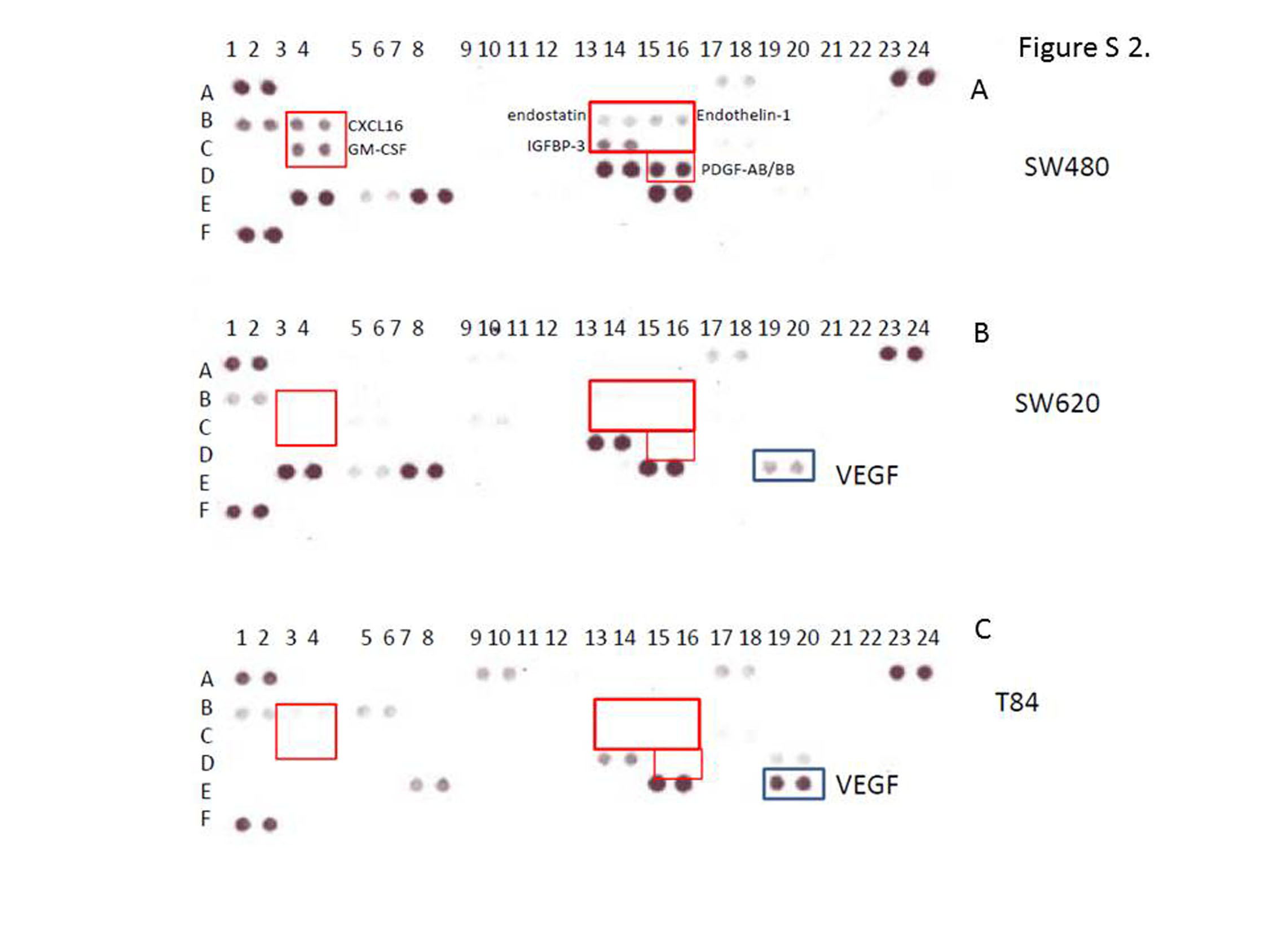

Supplement: S2 Fig — To identify common proteins that were upregulated or downregulated selectively in metastatic CRC cell lines, two metastatic CRC cell lines, SW620 and T84, their angiogenesis arrays were aligned with primary cell line SW480. Primary CRC cell line SW480 was used as a reference cell line, lymph-metastatic SW620 cell array was presented. In parallel, lung-metastatic T84 array was aligned. VEGF was upregulated in both SW620 and T84 cells. On the contrary, CXCL16, GM-CSF, endostatin, endothelin-1, IGFBP-3 and PDGF/AB, BB protein expression levels were decreased in both SW620 and T84 cells. (JPG) [file pone.0134948.s002.jpg]
