# Supplementary material for: Identification of Novel Biomarkers for Metastatic Colorectal Cancer Using Angiogenesis-Antibody Array and Intracellular Signaling Array
Source: PLoS One. 2015 Aug 10;10(8):e0134948. doi: 10.1371/journal.pone.0134948 (PMC4530953; doi:10.1371/journal.pone.0134948)
Supplement: S1 Table — 55 angiogenesis related proteins were presented in the S1 Table. The coordinates and target proteins were indicated together. (DOCX) [file pone.0134948.s003.docx]

**S1 Table. Coordinates of human angiogenesis array.**

| Coordinate | Target/control | Coordinate | Target/control |
| --- | --- | --- | --- |
| A1, A2 | Reference Spots | C17, C18 | IL-8 |
| A5, A6 | Activin A | C19, C20 | LAP (TGF-β1) |
| A7, A8 | ADAMTS-1 | C21, C22 | Leptin |
| A9, A10 | Angiogenin | C23, C24 | MCP-1 |
| A11, A12 | Angiopoietin-1 | D1, D2 | MIP-1α |
| A13, A14 | Angiopoietin-2 | D3, D4 | MMP-8 |
| A15, A16 | Angiostatin/Plasminogen | D5, D6 | MMP-9 |
| A17, A18 | Amphiregulin | D7, D8 | NRG1-β1 |
| A19, A20 | Artemin | D9, D10 | Pentraxin 3 (PTX3) |
| A23, A24 | Reference Spots | D11, D12 | PD-ECGF |
| B1, B2 | Coagulation Factor III | D13, D14 | PDGF-AA |
| B3, B4 | CXCL16 | D15, D16 | PDGF-AB/PDGF-BB |
| B5, B6 | DPPIV | D17, D18 | Persephin |
| B7, B8 | EGF | D19, D20 | Platelet Factor 4 (PF4) |
| B9, B10 | EG-VEGF | D21, D22 | PlGF |
| B11, B12 | Endoglin | D23, D24 | Prolactin |
| B13, B14 | Endostatin/Collagen XVIII | E1, E2 | Serpin B5 |
| B15, B16 | Endothelin-1 | E3, E4 | Serpin E1 |
| B17, B18 | FGF acidic | E5, E6 | Serpin F1 |
| B19, B20 | FGF basic | E7, E8 | TIMP-1 |
| B21, B22 | FGF-4 | E9, E10 | TIMP-4 |
| B23, B24 | FGF-7 | E11, E12 | Thrombospondin-1 |
| C1, C2 | GDNF | E13, E14 | Thrombospondin-2 |
| C3, C4 | GM-CSF | E15, E16 | uPA |
| C5, C6 | HB-EGF | E17, E18 | Vasohibin |
| C7, C8 | HGF | E19, E20 | VEGF |
| C9, C10 | IGFBP-1 | E21, E22 | VEGF-C |
| C11, C12 | IGFBP-2 | F1, F2 | Reference Spots |
| C13, C14 | IGFBP-3 | F23, F24 | Negative Control |
| C15, C16 | IL-1β |  |  |
