# Supplementary material for: Identification of Novel Biomarkers for Metastatic Colorectal Cancer Using Angiogenesis-Antibody Array and Intracellular Signaling Array
Source: PLoS One. 2015 Aug 10;10(8):e0134948. doi: 10.1371/journal.pone.0134948 (PMC4530953; doi:10.1371/journal.pone.0134948)
Supplement: S2 Table — 18 Intracellular signaling array proteins were presented. The coordinates and the target proteins were matched and presented. (DOCX) [file pone.0134948.s004.docx]

**S2 Table. Coordinates of human intracellular signaling array.**

|  | **Target** | **Modification** |
| --- | --- | --- |
| 1 | Positive control | N/A |
| 2 | Negative control | N/A |
| 3 | ERK1/2 | Phosphorylation |
| 4 | Stat1 | Phosphorylation |
| 5 | Stat3 | Phosphorylation |
| 6 | Akt Thr308 | Phosphorylation |
| 7 | Akt Ser473 | Phosphorylation |
| 8 | AMPKa | Phosphorylation |
| 9 | S6 ribosomal protein | Phosphorylation |
| 10 | mTOR | Phosphorylation |
| 11 | HSP27 | Phosphorylation |
| 12 | Bad | Phosphorylation |
| 13 | P70 S6 kinase | Phosphorylation |
| 14 | PRAS40 | Phosphorylation |
| 15 | P53 | Phosphorylation |
| 16 | P38 | Phosphorylation |
| 17 | SAPK/JNK | Phosphorylation |
| 18 | PARP | Cleavage |
| 19 | Caspase-3 | Cleavage |
| 20 | GSK-3b | Phosphorylation |
