# Supplementary material for: Identification of Novel Biomarkers for Metastatic Colorectal Cancer Using Angiogenesis-Antibody Array and Intracellular Signaling Array
Source: PLoS One. 2015 Aug 10;10(8):e0134948. doi: 10.1371/journal.pone.0134948 (PMC4530953; doi:10.1371/journal.pone.0134948)
Supplement: S3 Table — The human receptor tyrosine kinase proteins were presented. The coordinates and target proteins were indicated in the table. (DOCX) [file pone.0134948.s005.docx]

**S3 Table. Coordinates of human phosphor-receptor tyrosine kinase array.**

| coordinate | receptor family | rTK/control | coodinate | receptor family | rTK/control |
| --- | --- | --- | --- | --- | --- |
| A1, A2 | Reference Spots |  | D1, D2 | Tie | Tie-2 |
| A23, A24 | Reference Spots |  | D3, D4 | NGF R | TrkA |
| B1, B2 | EGF R | EGF R | D5, D6 | NGF R | TrkB |
| B3, B4 | EGF R | ErbB2 | D7, D8 | NGF R | TrkC |
| B5, B6 | EGF R | ErbB3 | D9, D10 | VEGF R | VEGF R1 |
| B7, B8 | EGF R | ErbB4 | D11, D12 | VEGF R | VEGF R2 |
| B9, B10 | FGF R | FGF R1 | D13, D14 | VEGF R | VEGF R3 |
| B11, B12 | FGF R | FGF R2α | D15, D16 | MuSK | MuSK |
| B13, B14 | FGF R | FGF R3 | D17, D18 | Eph R | EphA1 |
| B15, B16 | FGF R | FGF R4 | D19, D20 | Eph R | EphA2 |
| B17, B18 | Insulin R | Insulin R | D21, D22 | Eph R | EphA3 |
| B19, B20 | Insulin R | IGF-I R | D23, D24 | Eph R | EphA4 |
| B21, B22 | Axl | Axl | E1, E2 | Eph R | EphA6 |
| B23, B24 | Axl | Dtk | E3, E4 | Eph R | EphA7 |
| C1, C2 | Axl | Mer | E5, E6 | Eph R | EphB1 |
| C3, C4 | HGF R | HGF R | E7, E8 | Eph R | EphB2 |
| C5, C6 | HGF R | MSP R | E9, E10 | Eph R | EphB4 |
| C7, C8 | PDGF R | PDGF Rα | E11, E12 | Eph R | EphB6 |
| C9, C10 | PDGF R | PDGF Rβ | E13, E14 | Insulin R | ALK |
| C11, C12 | PDGF R | SCF R | E15, E16 |  | DDR1 |
| C13, C14 | PDGF R | Flt-3 | E17, E18 |  | DDR2 |
| C15, C16 | PDGF R | M-CSF R | E19, E20 | Eph R | EphA5 |
| C17, C18 | RET | c-Ret | E21, E22 | Eph R | EphA10 |
| C19, C20 | ROR | ROR1 | F1, F2 | Reference Spots |  |
| C21, C22 | ROR | ROR2 | F5, F6 | Eph R | EphB3 |
| C23, C24 | Tie | Tie-1 | F7, F8 |  | RYK |
|  | | | F23, F24 | Control (-) | PBS |
